# Supplementary figures and images for: Human immunodeficiency virus-1 induces host genomic R-loops and preferentially integrates its genome near the R-loop regions
Source: eLife. 2024 Dec 3;13:RP97348. doi: 10.7554/eLife.97348 (PMC11616997; doi:10.7554/eLife.97348)

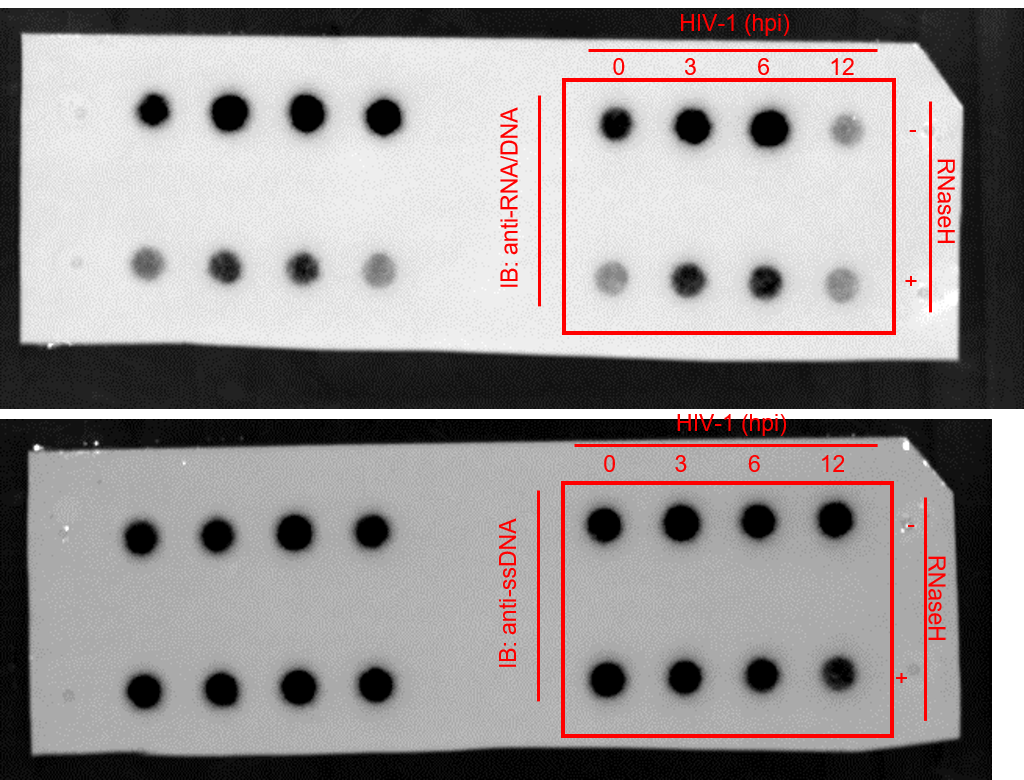

Supplement: Figure 1—source data 1. [file elife-97348-fig1-data1.zip › Figure 1D-labelled.png]

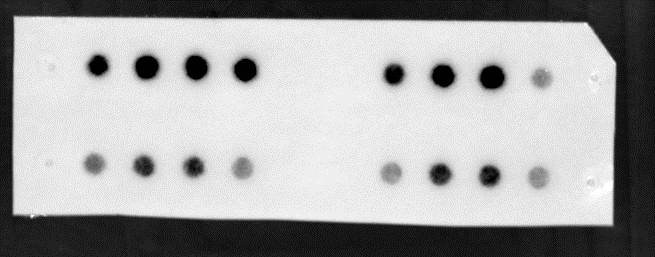

Supplement: Figure 1—source data 2. [file elife-97348-fig1-data2.zip › Figure 1D-1.png]

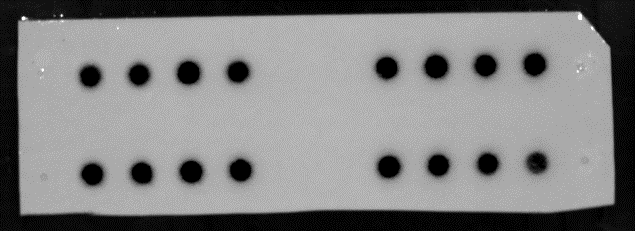

Supplement: Figure 1—source data 2. [file elife-97348-fig1-data2.zip › Figure 1D-2.png]

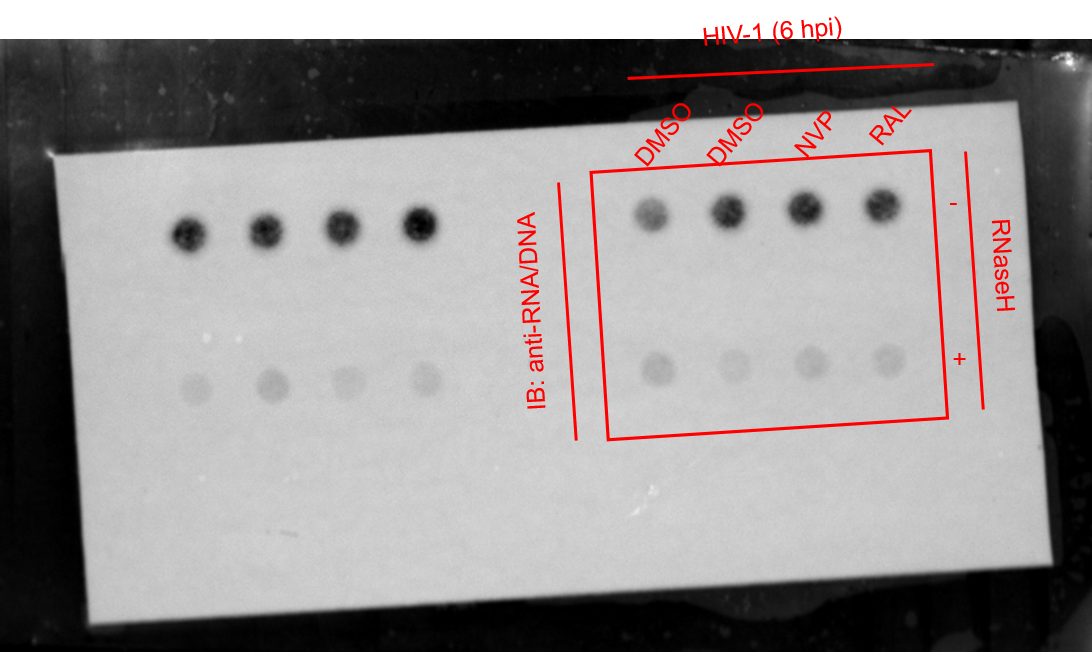

Supplement: Figure 1—figure supplement 6—source data 1. [file elife-97348-fig1-figsupp6-data1.zip › Figure1-Figure supplement 6B-RNADNA-lablled.png]

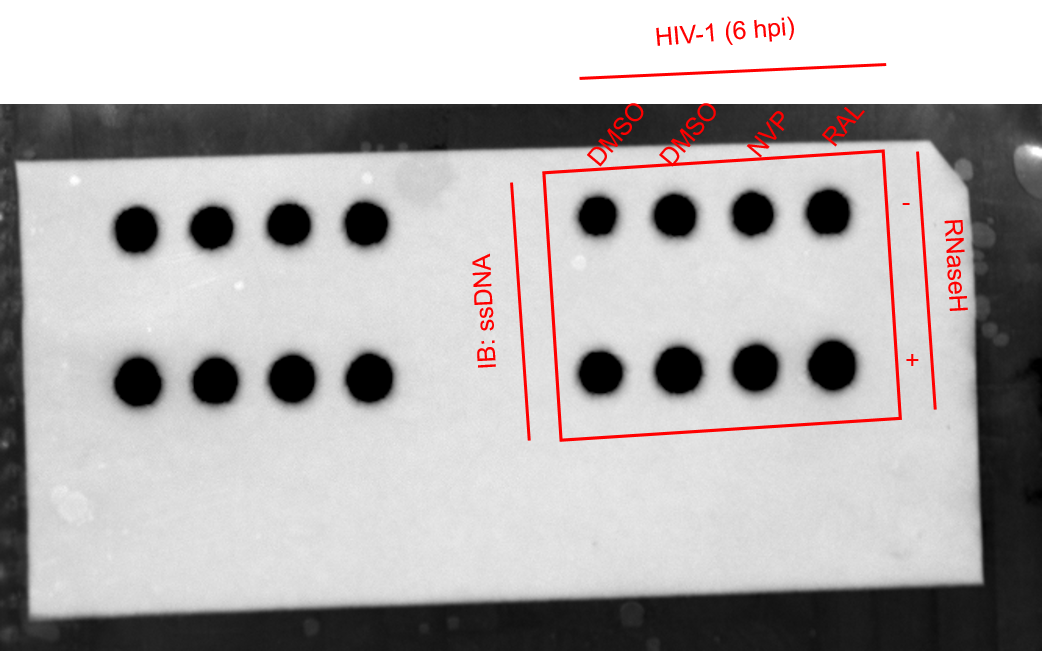

Supplement: Figure 1—figure supplement 6—source data 1. [file elife-97348-fig1-figsupp6-data1.zip › Figure1-Figure supplement 6B-dsDNA-lablled.png]

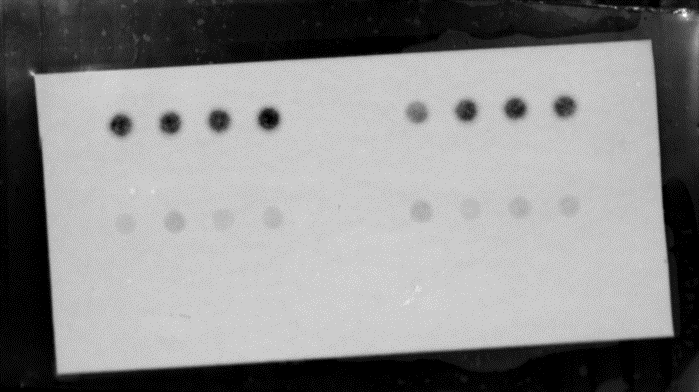

Supplement: Figure 1—figure supplement 6—source data 2. [file elife-97348-fig1-figsupp6-data2.zip › Figure1-Figure supplement 6B-RNADNA.png]

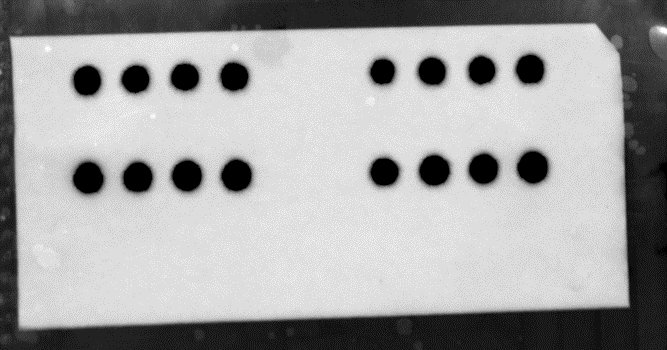

Supplement: Figure 1—figure supplement 6—source data 2. [file elife-97348-fig1-figsupp6-data2.zip › Figure1-Figure supplement 6B-dsDNA.png]

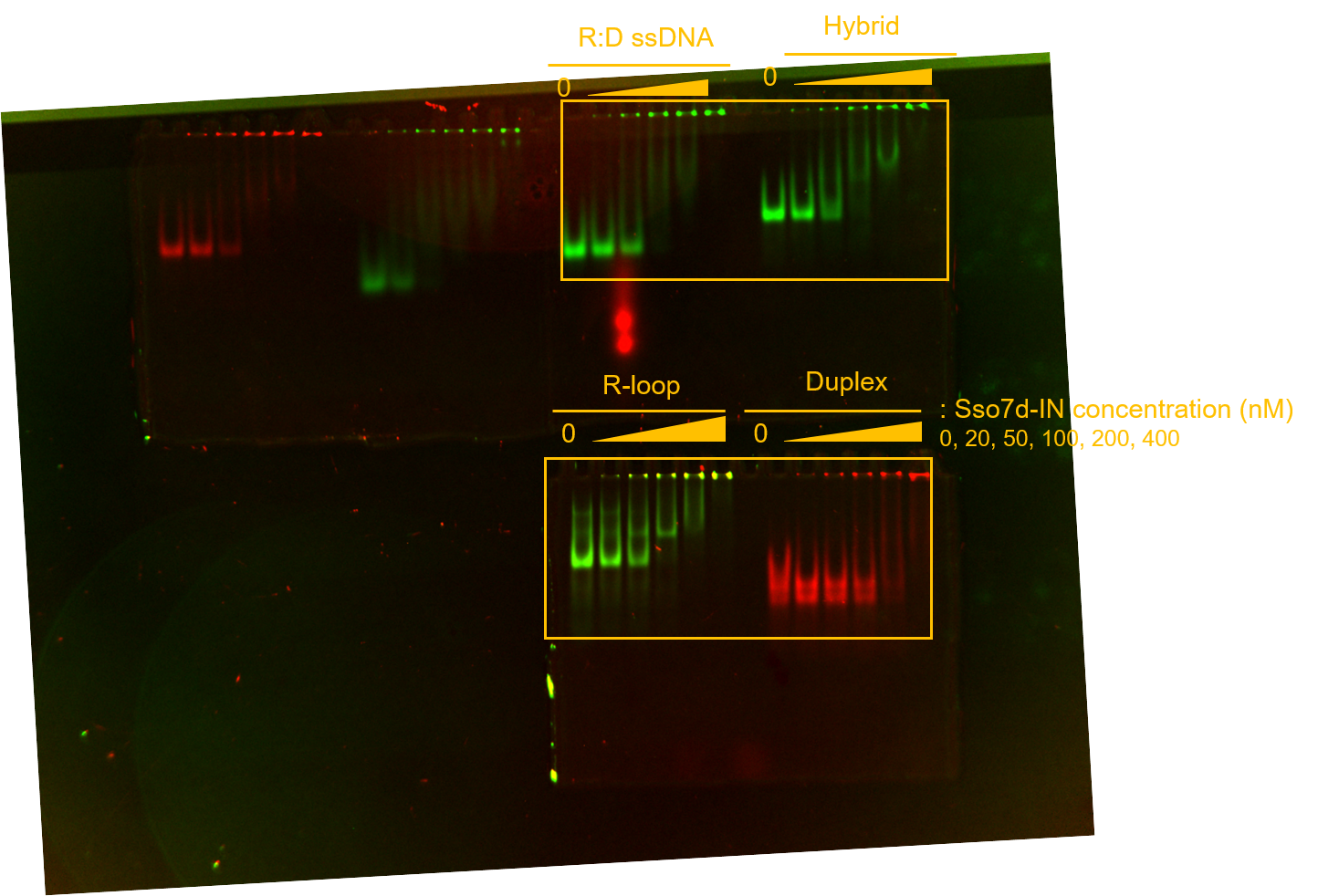

Supplement: Figure 5—source data 1. [file elife-97348-fig5-data1.zip › Figure 5A-labelled.png]

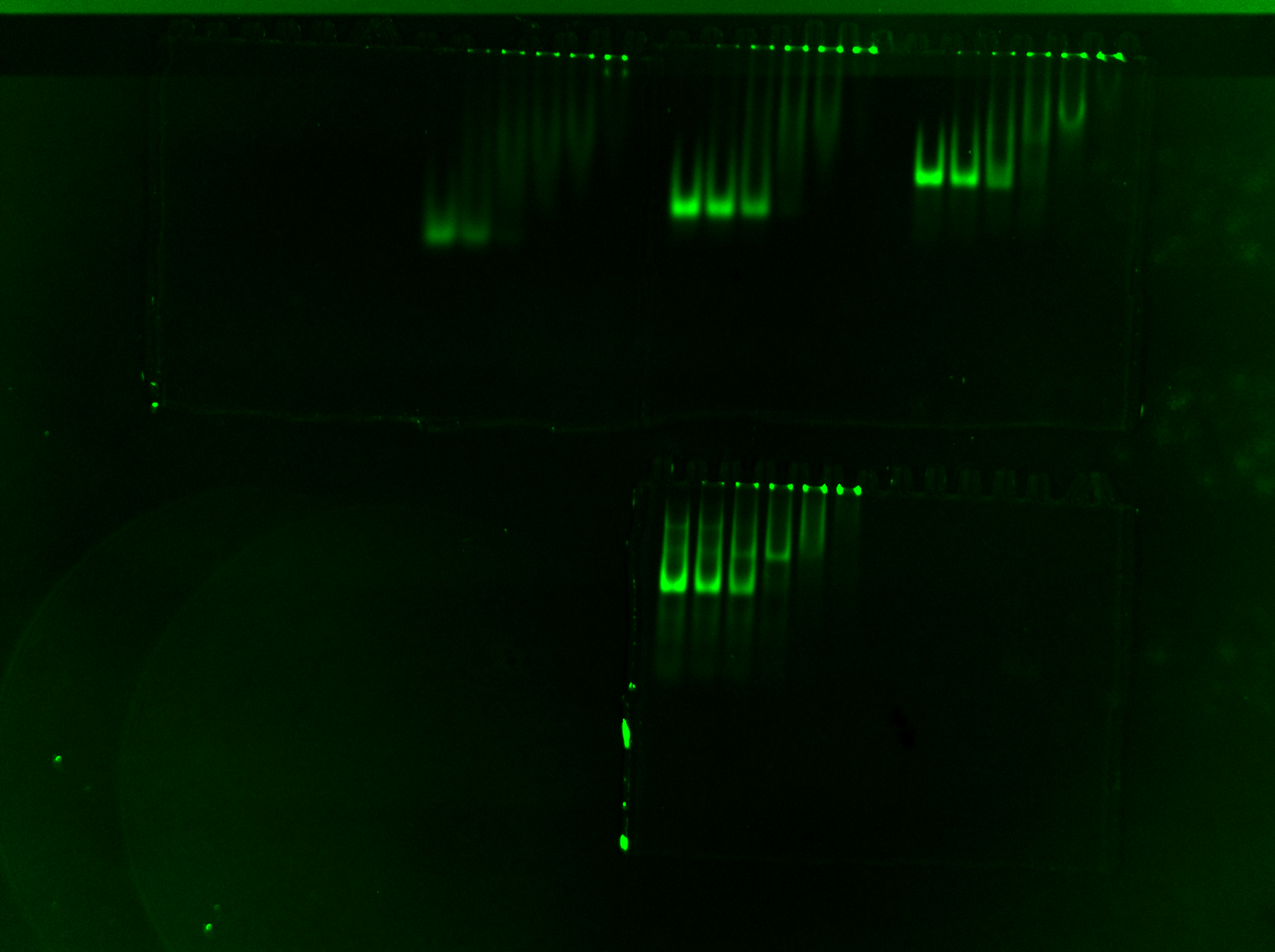

Supplement: Figure 5—source data 2. [file elife-97348-fig5-data2.zip › Figure 5A-Cy3.tif]

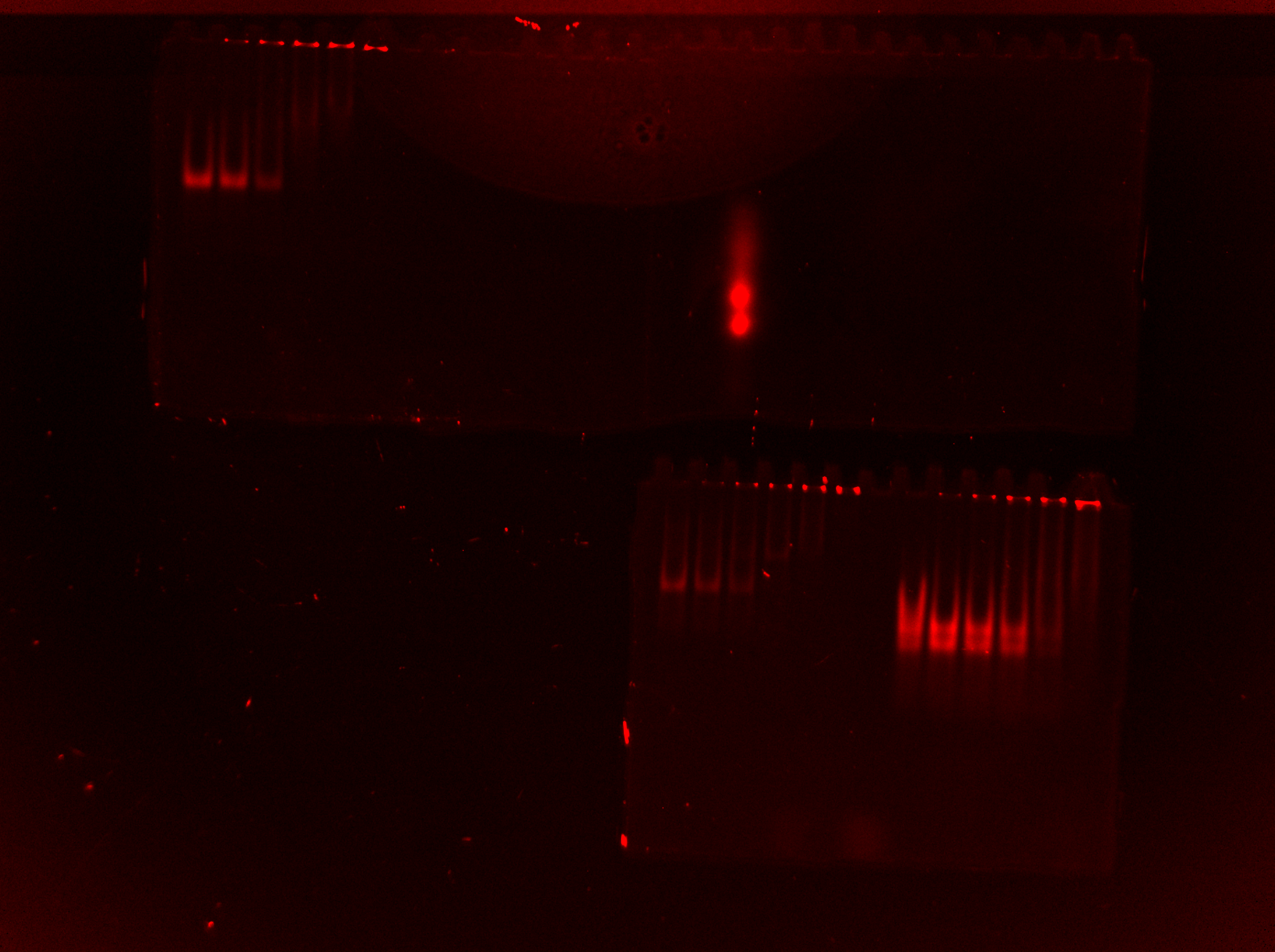

Supplement: Figure 5—source data 2. [file elife-97348-fig5-data2.zip › Figure 5A-Cy5.tif]

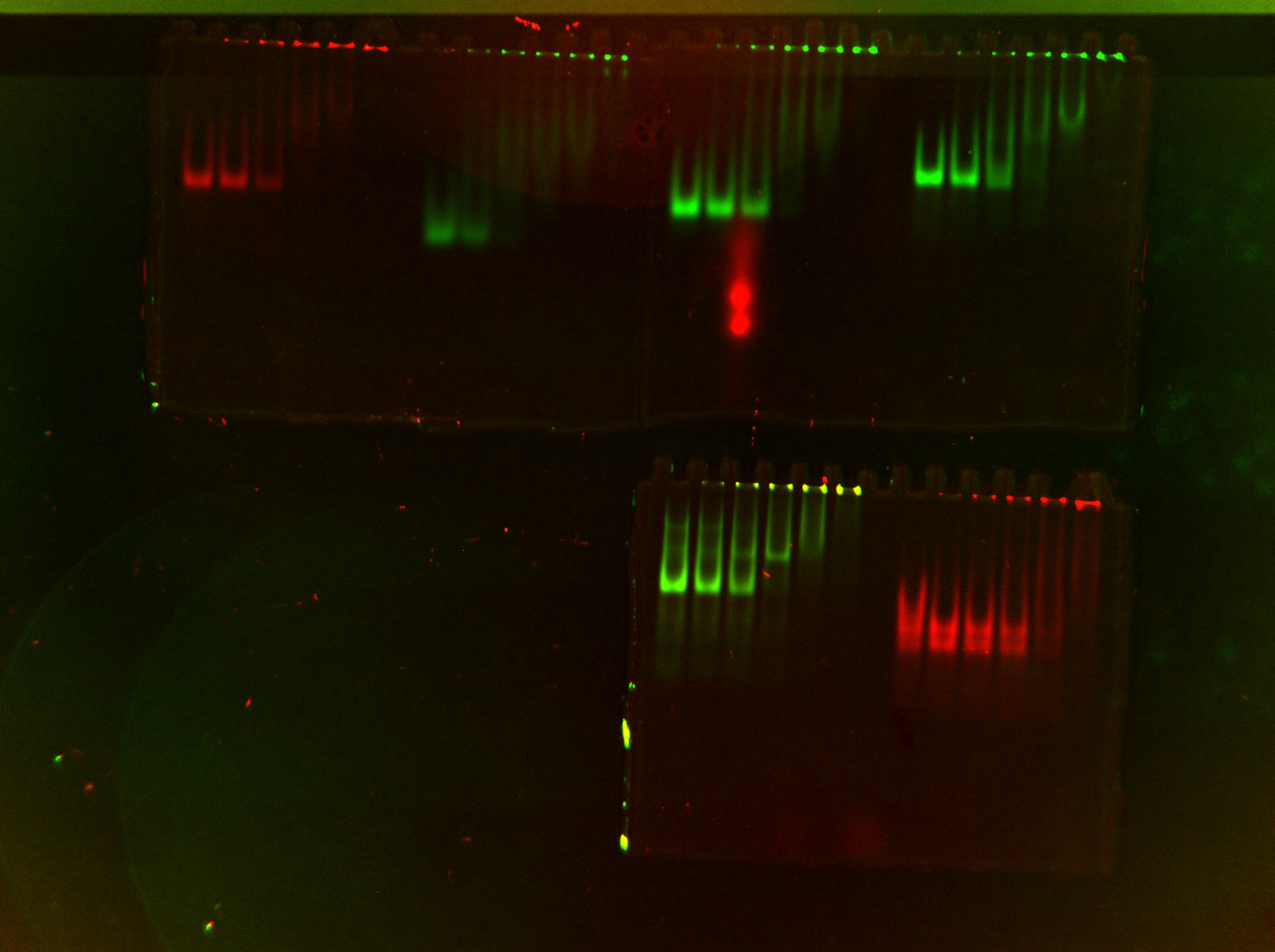

Supplement: Figure 5—source data 2. [file elife-97348-fig5-data2.zip › Figure 5A-merge.tif]

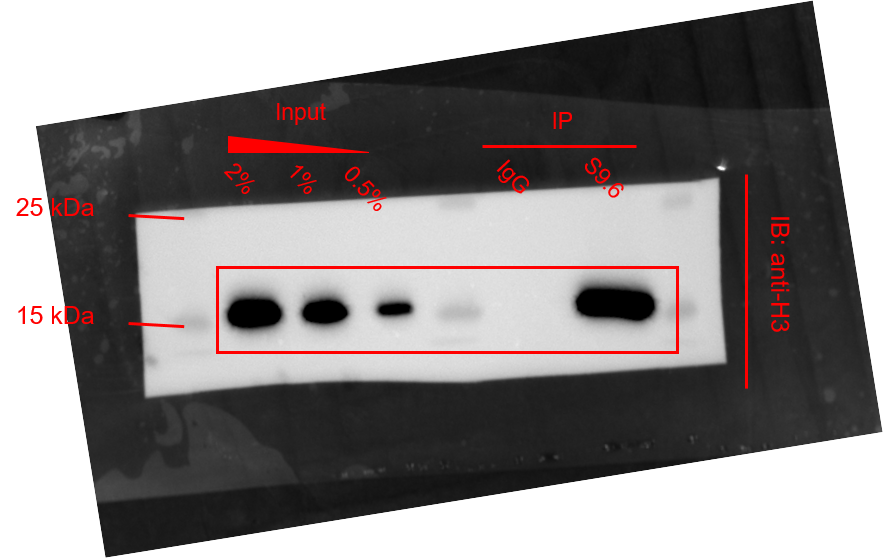

Supplement: Figure 5—source data 3. [file elife-97348-fig5-data3.zip › Figure 5C-H3-labelled.png]

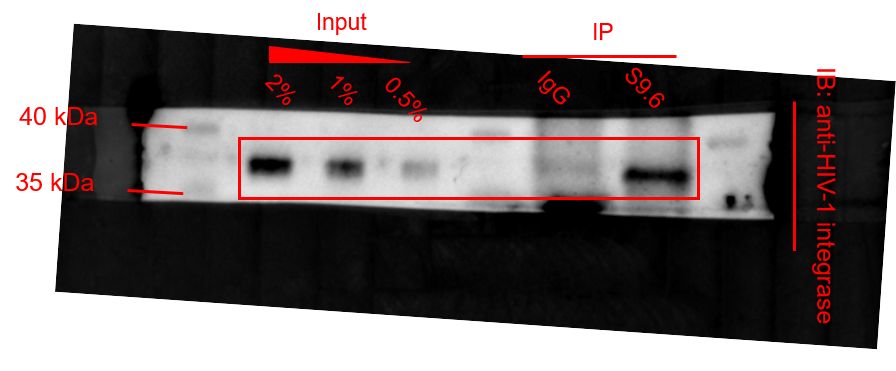

Supplement: Figure 5—source data 3. [file elife-97348-fig5-data3.zip › Figure 5C-HIV-1-integrase-labelled.png]

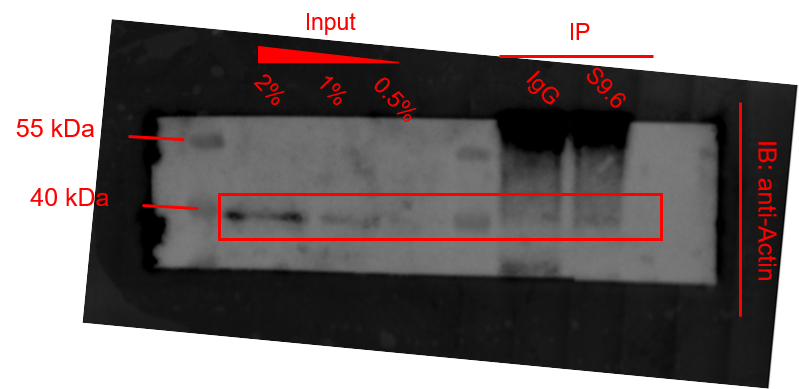

Supplement: Figure 5—source data 3. [file elife-97348-fig5-data3.zip › Figure 5C-actin-labelled.png]

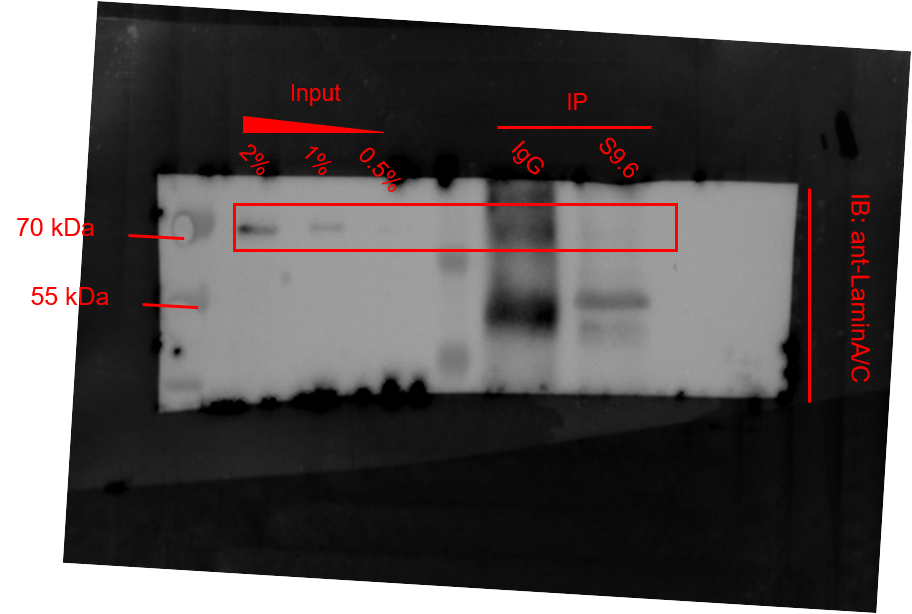

Supplement: Figure 5—source data 3. [file elife-97348-fig5-data3.zip › Figure 5C-laminAC-labelled.png]

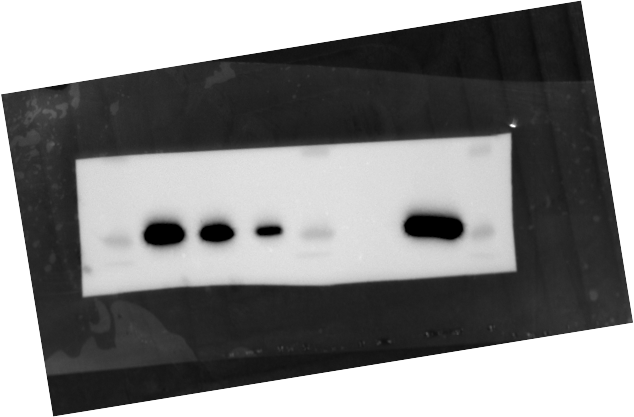

Supplement: Figure 5—source data 4. [file elife-97348-fig5-data4.zip › Figure 5C-H3.png]

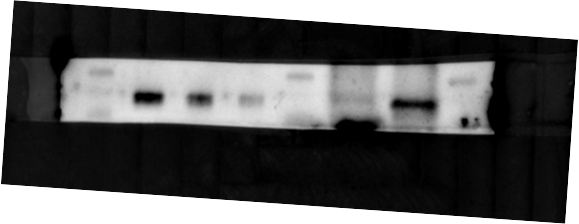

Supplement: Figure 5—source data 4. [file elife-97348-fig5-data4.zip › Figure 5C-HIV-1-integrase.png]

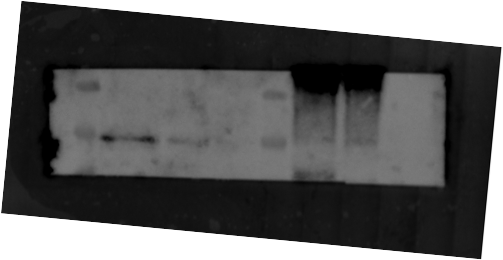

Supplement: Figure 5—source data 4. [file elife-97348-fig5-data4.zip › Figure 5C-actin.png]

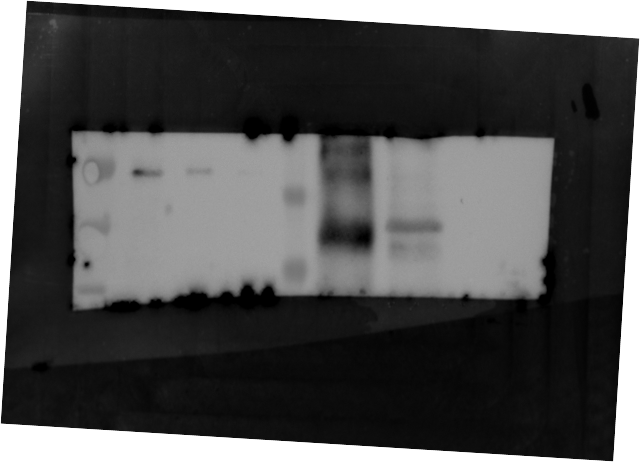

Supplement: Figure 5—source data 4. [file elife-97348-fig5-data4.zip › Figure 5C-laminAC.png]

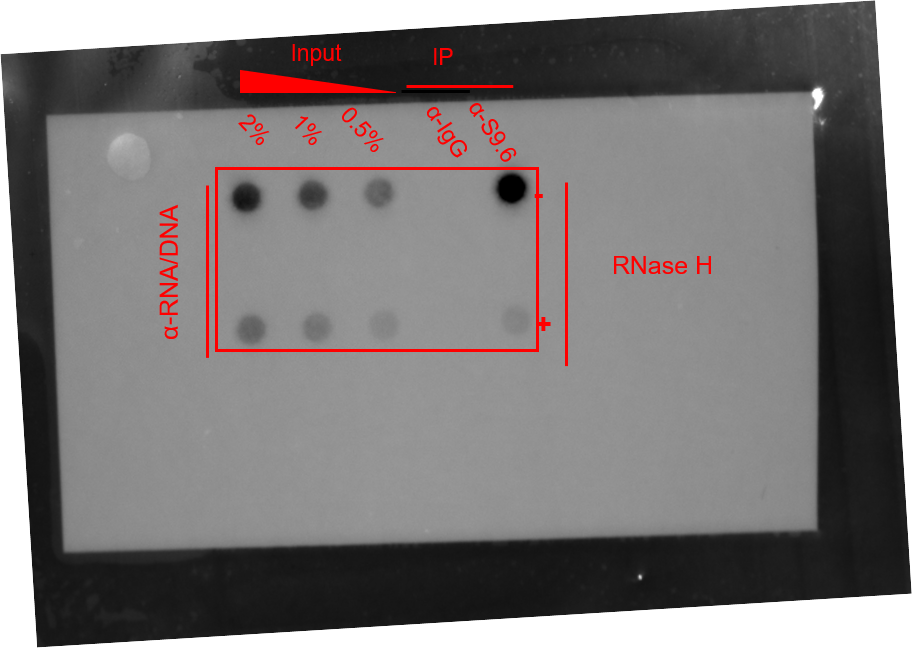

Supplement: Figure 5—source data 5. [file elife-97348-fig5-data5.zip › Figure 5D-labelled.png]

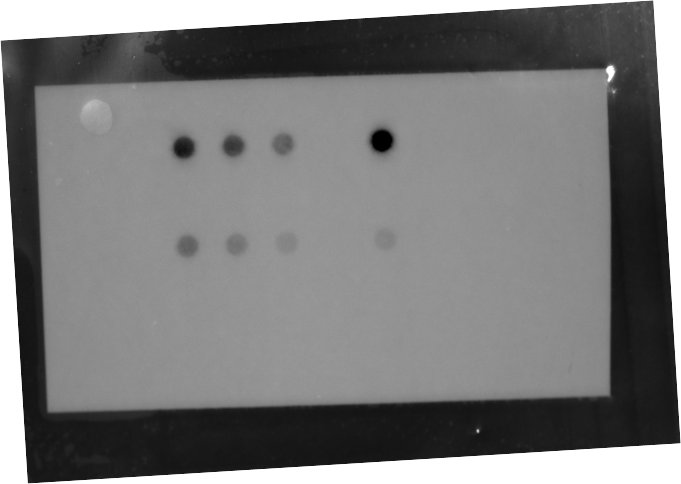

Supplement: Figure 5—source data 6. [file elife-97348-fig5-data6.zip › Figure 5D.png]

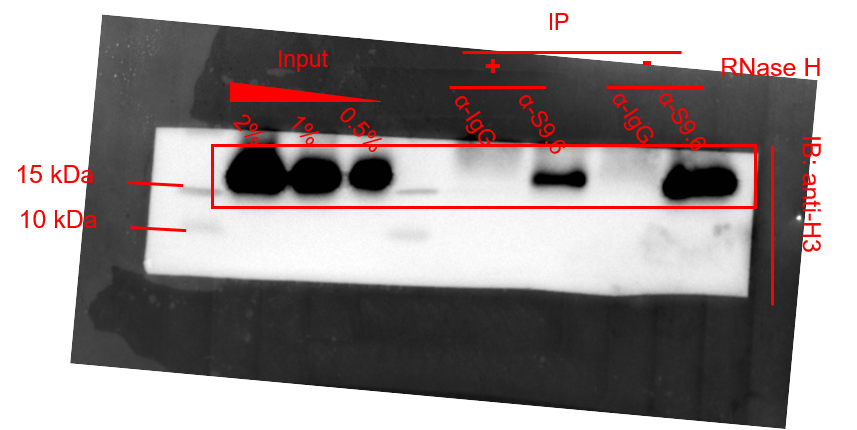

Supplement: Figure 5—source data 7. [file elife-97348-fig5-data7.zip › Figure 5E-H3-labelled.png]

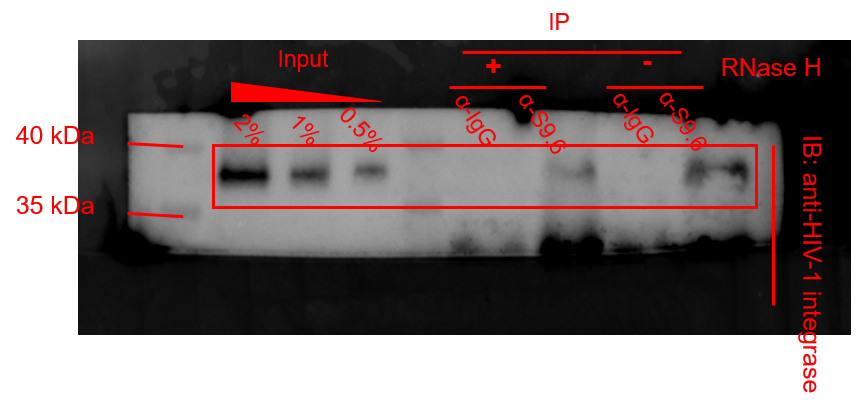

Supplement: Figure 5—source data 7. [file elife-97348-fig5-data7.zip › Figure 5E-HIV-1-integrase-labelled.png]

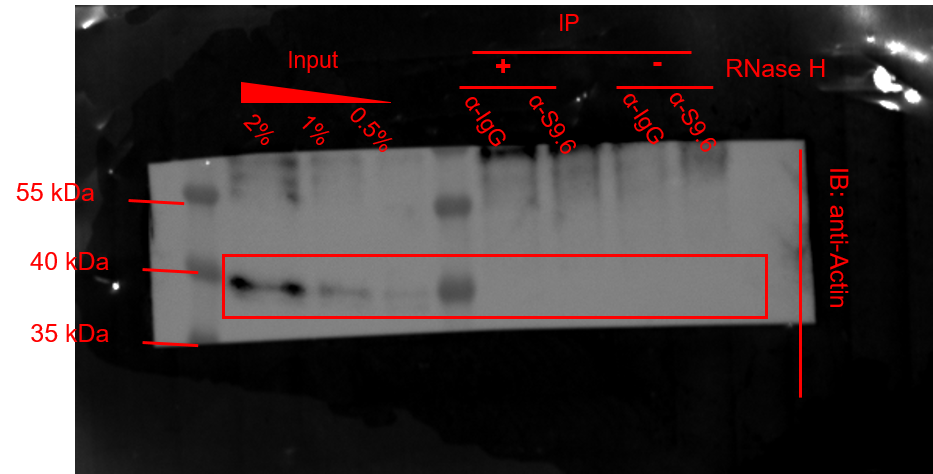

Supplement: Figure 5—source data 7. [file elife-97348-fig5-data7.zip › Figure 5E-actin-labelled.png]

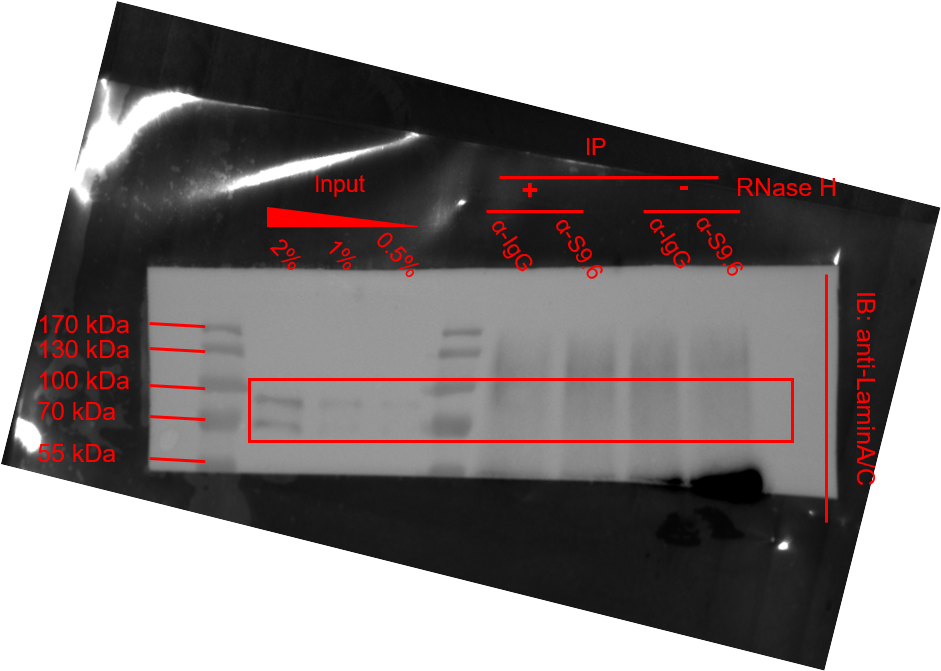

Supplement: Figure 5—source data 7. [file elife-97348-fig5-data7.zip › Figure 5E-laminAC-labelled.png]

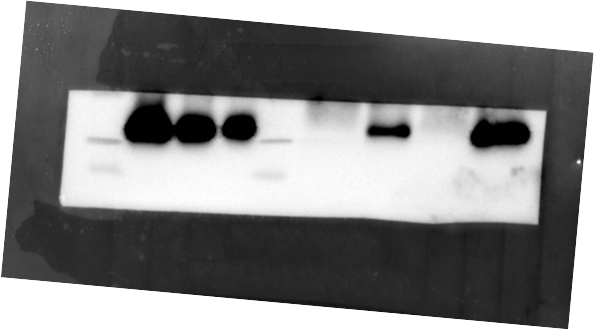

Supplement: Figure 5—source data 8. [file elife-97348-fig5-data8.zip › Figure 5E-H3.png]

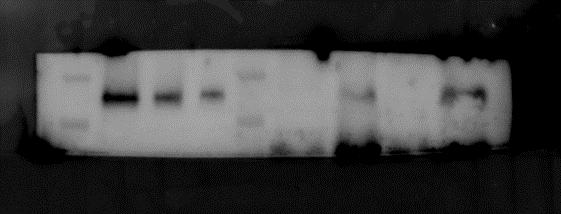

Supplement: Figure 5—source data 8. [file elife-97348-fig5-data8.zip › Figure 5E-HIV-1-integrase.png]

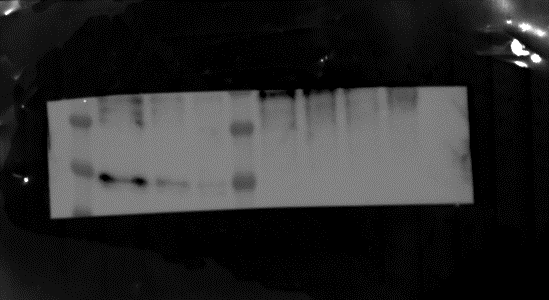

Supplement: Figure 5—source data 8. [file elife-97348-fig5-data8.zip › Figure 5E-actin.png]

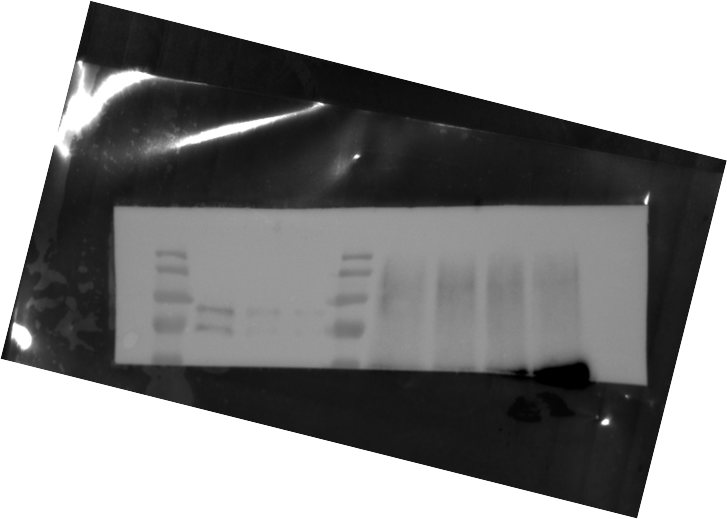

Supplement: Figure 5—source data 8. [file elife-97348-fig5-data8.zip › Figure 5E-laminAC.png]

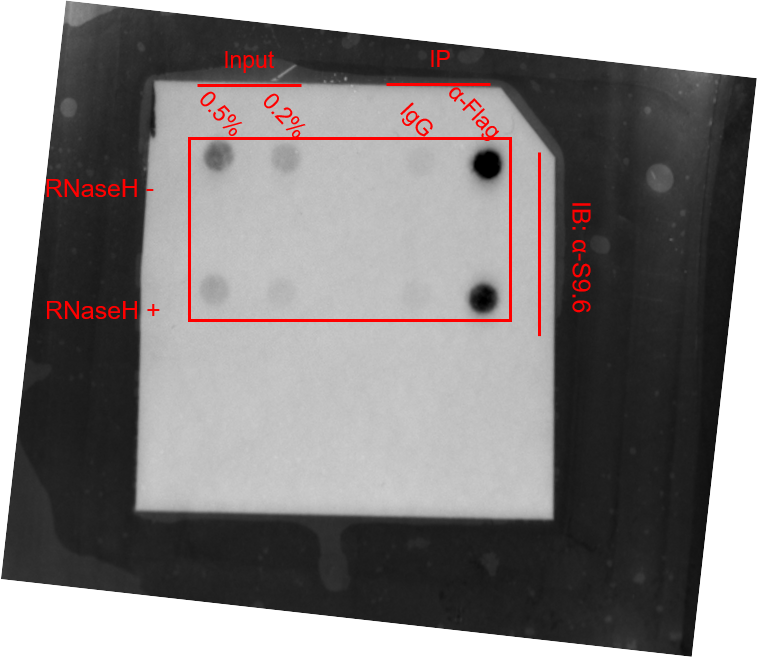

Supplement: Figure 5—source data 9. [file elife-97348-fig5-data9.zip › Figure 5F-labelled.png]

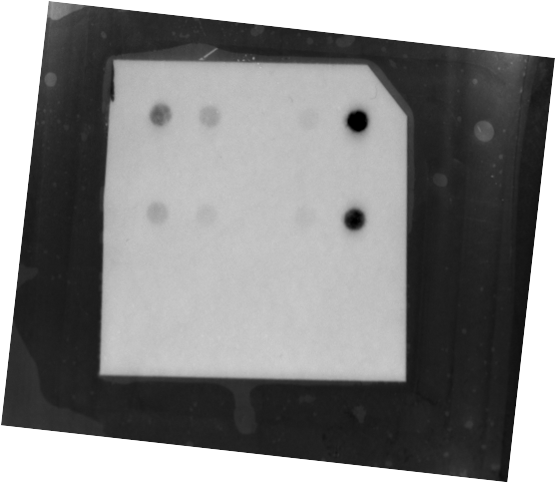

Supplement: Figure 5—source data 10. [file elife-97348-fig5-data10.zip › Figure 5F.png]

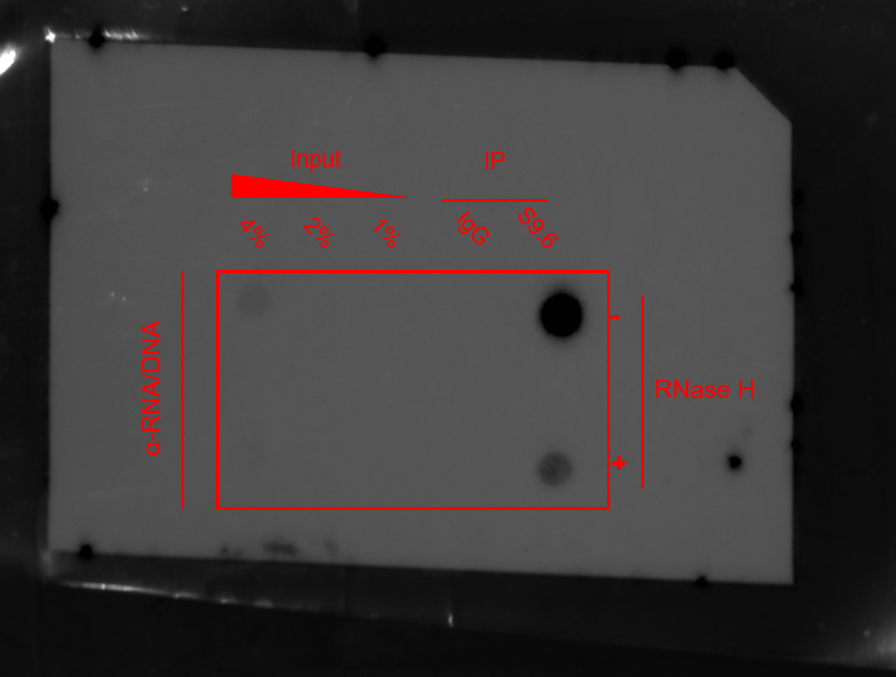

Supplement: Figure 5—figure supplement 1—source data 1. [file elife-97348-fig5-figsupp1-data1.zip › Figure 5-Figure supplement 1B-labelled.png]

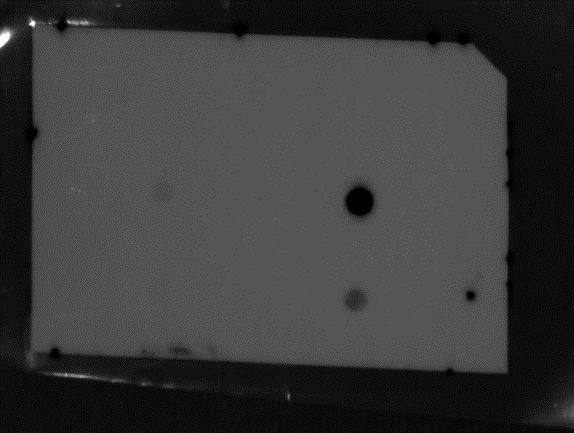

Supplement: Figure 5—figure supplement 1—source data 2. [file elife-97348-fig5-figsupp1-data2.zip › Figure 5-Figure supplement 1B.png]

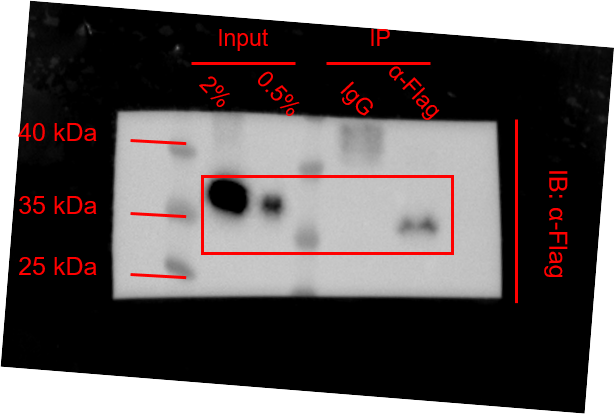

Supplement: Figure 5—figure supplement 1—source data 3. [file elife-97348-fig5-figsupp1-data3.zip › Figure 5-Figure supplement 1D Flag-labelled.png]

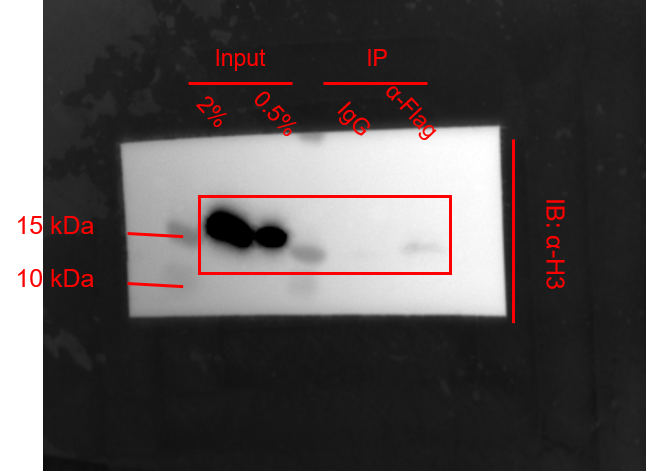

Supplement: Figure 5—figure supplement 1—source data 3. [file elife-97348-fig5-figsupp1-data3.zip › Figure 5-Figure supplement 1D H3-labelled.png]

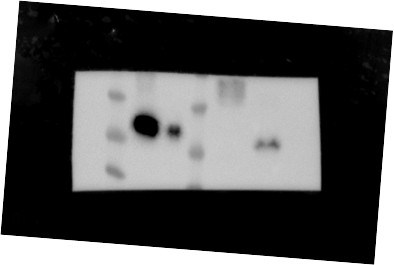

Supplement: Figure 5—figure supplement 1—source data 4. [file elife-97348-fig5-figsupp1-data4.zip › Figure 5-Figure supplement 1D Flag.png]

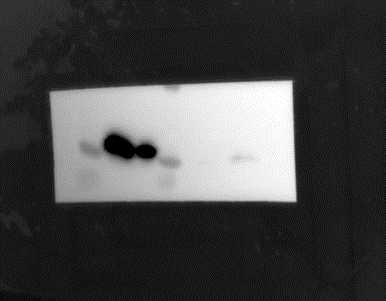

Supplement: Figure 5—figure supplement 1—source data 4. [file elife-97348-fig5-figsupp1-data4.zip › Figure 5-Figure supplement 1D H3.png]
